# Development of post-pericardiotomy syndrome is preceded by an increase in pro-inflammatory and a decrease in anti-inflammatory serological markers

**DOI:** 10.1186/1749-8090-7-72

**Published:** 2012-07-23

**Authors:** Nora Snefjellå, Knut Tore Lappegård

**Affiliations:** 1 University of Tromsø, Tromsø, Norway; 2 Coronary Care Unit, Division of Internal Medicine, Nordland Hospital, Bodø, Norway; 3 Institute of Clinical Medicine, University of Tromsø, Tromsø, Norway

**Keywords:** Post-pericardiotomy syndrome, inflammation, cytokines, chemokines

## Abstract

The post-pericardiotomy syndrome (PPS) is a common complication after cardiac surgery, occuring in 10-40% of patients. PPS may prolong hospitalization, and even serious complications like tamponade and constrictive pericarditis may occur. Early diagnosis and treatment may reduce morbidity. In 50 patients transferred to our hospital after cardiac surgery we found an increase in pro-inflammatory and a decrease in anti-inflammatory cytokines at admission in the patients later developing PPS compared to the patients who did not develop PPS. If confirmed in larger studies, these findings may prove useful in early identification of and targeted treatment in patients developing PPS.

## Findings

### Methods

We screened 50 consecutive post-operative coronary by-pass patients with a panel of inflammatory markers (cytokines, chemokines, growth factors and complement activation products [1]) as well as routine laboratory tests, and compared the values in patients developing and not developing PPS. PPS was defined according to usual practice in our department as a combination of fever unresponsive to antibiotics and pericardial effusion. Screening was performed the morning after transferral from a tertiary centre, usually 3–5 days after surgery, and none of the 50 patients had been diagnosed with PPS before transferral. Plasma samples were analyzed in batch when the last patient had been discharged from the hospital. The study was approved by the regional ethics committee.

### Results

**Figure 1:**
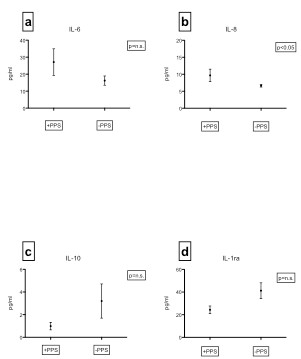
**Plasma levels at admission (3–5 days after surgery) of interleukin-6 (a), interleukin-8 (b), interleukin-10 (c) and interleukin-1 receptor antagonist (d) in 50 patients who did (+PPS, n = 11) or did not (−PPS, n = 39) develop post-pericardiotomy syndrome.** Mean and standard error of the mean.

PPS was diagnosed in 11 out of 50 patients (22%). The PPS patients were younger (64,9 vs 70,2 years, p < 0.05) and had higher levels of C-reactive protein (CRP) at admission (119 vs 90 mg/dL, p < 0.005) than those not developing PPS. Furthermore, at transferral to our hospital, serum levels of the pro-inflammatory cytokines interleukin (IL) -6 and IL-8 were higher and levels of the anti-inflammatory cytokines IL-10 and IL-1 receptor antagonist (IL-1ra) were lower in the PPS group, although this was statistically significant only for IL-8 (p < 0.05) (Figure 1). For the remainder of inflammatory markers there were no or only minor differences between the groups.

### Comment

Although usually benign, the occurence of PPS regularly prolongs hospitalization and in rare cases is associated with development of serious complications such as cardiac tamponade or constrictive pericarditis. Recently, colchicine was shown to be of benefit in preventing PPS [2], but as only a limited number of the patients undergoing surgery develop PPS, treating everyone may seem too aggressive. Early diagnosis and treatment reduces hospital stay, but current diagnostic criteria often include fever lasting more than one week or an inflammatory reaction unresponsive to antibiotic - resulting in delayed diagnosis. Serological markers could be helpful in identifying patients likely to develop the syndrome and thus allow early administration of targeted treatment. It is generally believed that PPS is associated with an inflammatory process although the culprit or antigen is less well defined.

In the pilot study presented here we show that patients developing PPS seem to have a shift in levels of inflammatory mediators in the days preceding development of a cardiac effusion – with higher levels of pro-inflammatory and lower levels of anti-inflammatory cytokines. The trend was clear, and the lack of statistical significance for some of the mediators may be due to the low number of participants, an obvious limitation of our study. Another limitation is the fact that screening samples were collected 3–5 days post-operatively and not at fixed time points. Although the patients developing PPS also had relatively higher levels of CRP, this finding is unspecific and thus of less use in diagnosing PPS. The lower age among the PPS-patients may indicate a relative immune paralysis in the older patients, reducing the risk of developing PPS. We suggest that our findings should be confirmed in a larger trial as they may serve as a tool for early detection and treatment of PPS.

## Abbreviations

PPS, Post-pericardiotomy syndrome; CRP, C-reactive protein; IL, Interleukin; Ra, Receptor antagonist.

## Competing interests

The authors report no competing interests.

## Authors’ contributions

NS and KTL designed the study, KTL drafted the manuscript and both authors read and approved the final manuscript.
